# Serum and Plasma Zinc and Copper Levels in Oral Leukoplakia: A Systematic Review and Meta-Analysis

**DOI:** 10.3390/antiox15101312

**Published:** 2026-10-12

**Authors:** Patryk Wiśniewski, Monika Stępniewska, Piotr Melion, Zuzanna Poloczek, Maksymilian Pawluczuk, Dawid Chodziński, Magdalena Sulewska, Małgorzata Pietruska

**Affiliations:** 1 Department of Periodontal and Oral Mucosa Diseases, Medical University of Białystok, ul. Waszyngtona 13, 15-269 Bialystok, Poland; magdalena.sulewska@umb.edu.pl (M.S.); malgorzata.pietruska@umb.edu.pl (M.P.); 2 Students’ Research Group on Oral Mucosal Diseases, Department of Periodontal and Oral Mucosa Diseases, Medical University of Białystok, ul. Waszyngtona 13, 15-269 Bialystok, Poland; 45029@student.umb.edu.pl (M.S.); 45017@student.umb.edu.pl (P.M.); 45025@student.umb.edu.pl (Z.P.); 45020@student.umb.edu.pl (M.P.); 44989@student.umb.edu.pl (D.C.)

**Keywords:** oral leukoplakia, zinc, copper, trace elements, oxidative stress, oral potentially malignant disorders, biomarkers

## Abstract

Background: Oral leukoplakia (OL) is a common oral potentially malignant disorder in which oxidative imbalance and altered trace-element homeostasis may contribute to pathogenesis. This systematic review and meta-analysis evaluated serum/plasma zinc and copper concentrations in patients with OL versus healthy controls. Materials and Methods: Following PRISMA and Cochrane recommendations (PROSPERO: CDR420261427977), PubMed, Scopus, Web of Science, and Cochrane were searched for observational studies comparing circulating zinc and/or copper levels in OL and controls. Study quality was assessed using the Newcastle–Ottawa Scale and certainty of evidence using GRADE. Hedges’ g with 95% confidence intervals (CIs) was pooled. Results: Ten studies including 495 participants were analyzed. Zinc concentrations were significantly lower in OL (8 studies; 431 participants; g = −1.84, 95% CI: −3.25 to −0.43; *p* = 0.01; I^2^ = 96.85%), whereas copper concentrations were significantly higher (9 studies; 449 participants; g = 1.20, 95% CI: 0.70–1.70; *p* < 0.001; I^2^ = 81.90%). Sensitivity analyses confirmed the direction of both associations. Certainty of evidence was very low. Conclusions: Patients with OL showed lower circulating zinc and higher copper concentrations than healthy controls. Given the substantial heterogeneity and very low certainty of evidence, these findings should be interpreted as group-level differences only and do not establish a specific metabolic, pathogenic, causal, or diagnostic role of Zn or Cu in OL.

## 1. Introduction

Oral leukoplakia (OL) is one of the most common oral potentially malignant disorders (OPMDs) [1,2]. Its clinical significance primarily stems from the increased risk of progression to oral squamous cell carcinoma [3]. The global prevalence of OL has been estimated at 1.39%; however, it reaches 3.41% in population-based studies and is markedly higher in selected high-risk groups. According to the most recent comprehensive systematic review and meta-analysis, the pooled malignant transformation rate of OL is 7.2%, with the risk of progression depending, among other factors, on the clinical and histopathological characteristics of the lesion [3].

Despite the well-established effects of conventional risk factors, including tobacco smoking, alcohol consumption, and chronic exposure of the oral mucosa to irritants, the mechanisms underlying the development and progression of oral leukoplakia remain incompletely understood [4,5]. In this context, increasing attention has been paid to disturbances in oxidant–antioxidant balance, which may constitute one of the links between chronic exposure to damaging factors and the cellular alterations observed in potentially malignant lesions [6,7]. Excessive production of reactive oxygen species, accompanied by impaired antioxidant defense mechanisms, promotes oxidative damage to lipids, proteins, and genetic material, thereby potentially increasing cellular instability and facilitating the progression of premalignant lesions [8,9,10]. A recently published systematic review and meta-analysis demonstrated disturbances in salivary oxidant–antioxidant balance in patients with OL, including increased levels of oxidative damage markers and decreased levels of selected antioxidants [11].

Trace elements, including zinc and copper, are considered important contributors to the maintenance of oxidant–antioxidant balance [12,13,14]. As cofactors of numerous enzymes, including copper/zinc superoxide dismutase (Cu/Zn-SOD), they participate in the neutralization of reactive oxygen species and the protection of cells against oxidative damage [15,16,17,18,19,20]. Altered concentrations of these elements may lead to the dysregulation of redox homeostasis, thereby contributing to increased oxidative stress [21,22,23,24]. Owing to their important role in maintaining cellular homeostasis, zinc and copper are increasingly being investigated as potential biomarkers of premalignant and malignant oral lesions, including leukoplakia [24,25].

Despite growing interest in the role of zinc and copper in the pathogenesis of oral leukoplakia, the findings of studies evaluating their concentrations in biological materials, including serum and saliva, remain inconclusive. To the best of our knowledge, no comprehensive systematic review and meta-analysis has yet synthesized the available evidence regarding alterations in the concentrations of these elements in patients with oral leukoplakia.

The primary objective of this systematic review and meta-analysis was to comprehensively evaluate, both qualitatively and quantitatively, serum and plasma zinc and copper concentrations in patients with oral leukoplakia compared with healthy controls. The secondary objective was to investigate potential associations between the levels of the analyzed trace elements and the extent of oral lesions and, where sufficient data were available, to assess their correlations with other clinical and biological factors.

## 2. Materials and Methods

### 2.1. Search Strategy, Eligibility Criteria, and Data Extraction

The study protocol was registered with PROSPERO (CRD420261427977) and adhered to the Cochrane Handbook and PRISMA 2020 guidelines [26,27] (Supplementary File S1). The prespecified research question was: Do serum and plasma zinc and copper concentrations differ between individuals diagnosed with oral leukoplakia and healthy control subjects? Accordingly, the population of interest comprised individuals diagnosed with oral leukoplakia, the comparator group consisted of healthy individuals without oral leukoplakia, and the outcomes of interest were serum and plasma zinc and copper concentrations.

To identify all available evidence on serum/plasma trace element imbalances (zinc and copper) in oral leukoplakia, we conducted a systematic literature search across four electronic databases: PubMed, Scopus, Web of Science, and Cochrane. In addition to the electronic database search, backward citation searching was performed by manually screening the reference lists of potentially relevant studies identified during the screening process. This supplementary search identified four additional records, which were subsequently assessed using the same predefined eligibility criteria as records retrieved through the electronic database search.

1. For PubMed: (leukoplak* OR leucoplak* OR oral leukoplakia OR OLK OR OL) AND (trace elements OR biomarkers OR zinc OR copper OR copper/zinc ratio OR zinc level OR copper level) AND (serum OR plasma OR spectrometry OR spectrophotometry OR colorimetry)
2. For Scopus: (leukoplak* OR leucoplak* OR oral leukoplakia OR OLK OR OL) AND (trace elements OR biomarkers OR zinc OR copper OR copper/zinc ratio OR zinc level OR copper level) AND (serum OR plasma OR spectrometry OR spectrophotometry OR colorimetry)
3. For Web of Science: (leukoplak* OR leucoplak* OR “oral leukoplakia” OR “oral leukoplakias” OR OLK) AND (“trace element*” OR “trace metal*” OR micronutrient* OR biomarker* OR zinc OR copper OR “copper zinc ratio” OR “zinc copper ratio” OR “copper-to-zinc ratio” OR “zinc-to-copper ratio” OR “Cu Zn ratio” OR “Zn Cu ratio”) AND (serum OR plasma OR blood OR “whole blood”)
4. For Cochrane: (leukoplakia OR leucoplakia OR “oral leukoplakia” OR OLK OR OL) AND (“trace elements” OR biomarkers OR zinc OR copper OR “copper zinc ratio” OR “zinc level” OR “copper level”) AND (serum OR plasma OR spectrometry OR spectrophotometry OR colorimetry)

The first search was conducted on 3 July 2026, and the last on 14 July 2026. The objective was to identify observational investigations that evaluated serum or plasma trace elements—copper and zinc—in individuals diagnosed with oral leukoplakia (confirmed either clinically or through histopathological examination) relative to systemically healthy control subjects. No chronological limitations were imposed on publication dates; however, eligibility was restricted to English-language articles reporting on human participants. Exclusion criteria encompassed: studies using only saliva or alternative biological matrices, non-comparative study designs, case reports, reviews, conference abstracts, editorials, animal or in vitro experiments, and reports that failed to present sufficient quantitative data for extraction or meta-analytic synthesis.

Two reviewers (M.S. and Z.P.) independently evaluated all titles and abstracts, after which full-text articles were assessed against the prespecified PECO-based eligibility criteria (Table 1) governing the inclusion and exclusion of studies. Discrepancies between reviewers were resolved through discussion; in instances where agreement proved unattainable, a third reviewer (P.W.) was consulted for arbitration. Both reviewers (M.S. and Z.P.) utilized Zotero reference management software to identify and eliminate potential duplicate entries throughout the screening process.

For articles reporting insufficient quantitative data, we contacted the corresponding authors to seek additional information. Studies were excluded from the pooled analysis if we received no response or if the required data could not be supplied.

From each eligible study, we extracted:

1. article-level information—first author, year of publication, country, study design, and sample size;
2. participant characteristics—age and sex;
3. outcome data—serum/plasma trace elements reported as mean ± SD or median with IQR for both leukoplakia patients and controls, sample sizes, and details of the serum/plasma collection protocol and analytical methods.

### 2.2. Quality Assessment

The methodological quality of the included observational studies was independently evaluated by two reviewers (Z.P. and P.M.) using the modified Newcastle–Ottawa Scale (NOS), a validated tool for assessing the risk of bias in case–control studies. The scale evaluates three methodological domains: selection of study groups, comparability of cases and controls, and ascertainment of exposure [28]. Each study was awarded up to nine stars according to the predefined NOS criteria, with higher scores indicating better methodological quality.

Any disagreements between the reviewers were resolved through discussion until consensus was reached. If necessary, a third reviewer (P.W.) was consulted to resolve unresolved discrepancies. Following established recommendations, studies receiving 7–9 stars were classified as high quality with a low risk of bias, studies scoring 4–6 stars were considered of moderate quality, and studies with 0–3 stars were regarded as low-quality studies with a high risk of bias.

The overall certainty of evidence was assessed using the Grading of Recommendations Assessment, Development, and Evaluation (GRADE) approach [29].

### 2.3. Statistical Analysis

Quantitative synthesis was performed whenever at least two independent studies reported serum or plasma concentrations of zinc or copper in patients with oral leukoplakia and healthy control subjects. Effect estimates were calculated from the reported sample sizes together with the corresponding mean ± standard deviation (SD) values and were expressed as standardized mean differences (SMDs) with 95% confidence intervals (95% CIs), allowing direct comparison across studies that used different analytical methods or measurement scales. Statistical significance was established at a two-sided *p* value <0.05.

Between-study heterogeneity was evaluated using Cochran’s Q statistic and quantified with the I^2^ statistic. Heterogeneity was interpreted as low, moderate, or high for I^2^ values of approximately 25%, 50%, and 75%, respectively [30]. A random-effects model was adopted when substantial heterogeneity was detected (I^2^ > 50%), whereas a fixed-effect model was applied in analyses with lower heterogeneity.

In two studies, results were reported either separately by epithelial dysplasia grade or for broader OPMD groups that included lesions other than oral leukoplakia, rather than as a single estimate for the oral leukoplakia group. Therefore, only the oral leukoplakia-specific subgroups were combined to derive an overall value for patients with oral leukoplakia. To obtain a single estimate suitable for inclusion in the meta-analysis, the subgroups were combined using standard formulas for pooling independent groups. The combined sample size was calculated as the sum of the subgroup sample sizes, while the combined mean was calculated as a sample-size-weighted average of the subgroup means. The pooled SD was calculated by accounting for both the within-subgroup variance and the differences between subgroup means, according to the following formula:

(1) $$SD_{combined}=\sqrt{\frac{\sum (\left.n_{i}-1\right)SD_{i}^{2}+\sum n_{i}{\left({\bar{x}}_{i}-{\bar{x}}_{combined}\right)}^{2}}{\sum n_{i}-1}}$$

This procedure was applied separately to copper and zinc concentrations, where applicable, to derive one overall value for the oral leukoplakia group.

The robustness of the pooled estimates was examined by performing leave-one-out sensitivity analyses, in which each study was sequentially removed and the overall SMD was recalculated. Owing to the small number of studies available for each outcome, publication bias was not evaluated using funnel plots, in accordance with current methodological recommendations [31]. All statistical analyses were carried out using StataBe 19.5 (StataCorp LLC, College Station, TX, USA).

## 3. Results

### 3.1. Literature Searches

**Figure 1:**
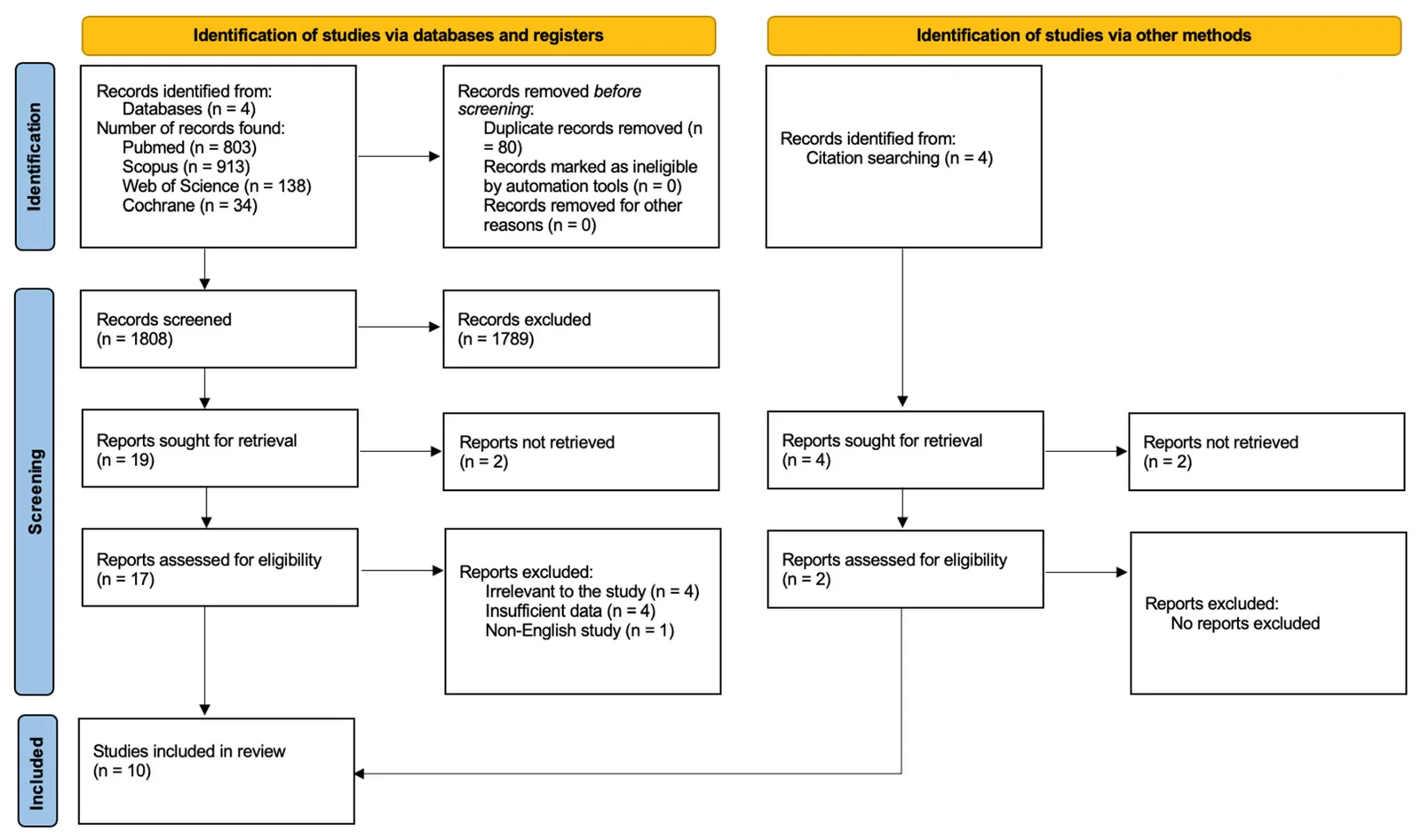
PRISMA flow chart. (Accessed on 21 July 2026).

The study selection process is summarized in the PRISMA flow diagram (Figure 1). The electronic search of PubMed, Scopus, Web of Science and Cochrane identified a total of 1888 records up to 14 July 2026 (803 from PubMed, 913 from Scopus, 138 from Web of Science, and 34 from Cochrane).

After removal of 80 duplicate records, 1808 unique records remained for title and abstract screening. Of these, 1789 were excluded as clearly irrelevant (e.g., studies not involving oral leukoplakia, absence of serum sampling, no assessment of trace elements, copper or/and zinc, animal or in vitro studies, reviews, or non-English publications).

Nineteen full-text articles were sought for retrieval. Two reports could not be obtained despite attempts to access the full text. Seventeen full-text articles were assessed for eligibility, and nine were excluded because they did not meet the predefined PECO criteria.

Backward citation searching of the reference lists of potentially relevant studies identified four additional records. These records were sought for retrieval, and two full-text articles were obtained and assessed for eligibility according to the predefined PECO criteria. Ultimately, ten studies fulfilled the inclusion criteria and were included in the qualitative synthesis [32,33,34,35,36,37,38,39,40,41] (Figure 1).

### 3.2. Study Characteristics

A total of ten eligible studies were identified and are detailed in Table 2. The total participant pool across these case–control investigations was 495 individuals, comprising 247 patients with oral leukoplakia and 248 systemically healthy control subjects. The trace elements investigated in each study comprised serum/plasma zinc, copper, or a combination of the two. A detailed account of the methodological considerations governing serum/plasma sampling and subsequent analytical procedures—sample volume, coagulation time, and pre-analytical processing—is presented in Table 3. The table also summarizes the diagnostic criteria used to define OL in the included studies, including whether the diagnosis was based on clinical assessment and whether histopathological evaluation was performed.

Table 4 presents a comprehensive summary of the meta-analysis findings for each trace element, detailing the number of studies and participants contributing to each pooled estimate, the standardized mean difference with accompanying 95% confidence intervals, and the corresponding heterogeneity metrics (Q statistic, *p* value, and I^2^ value).

**Figure 2:**
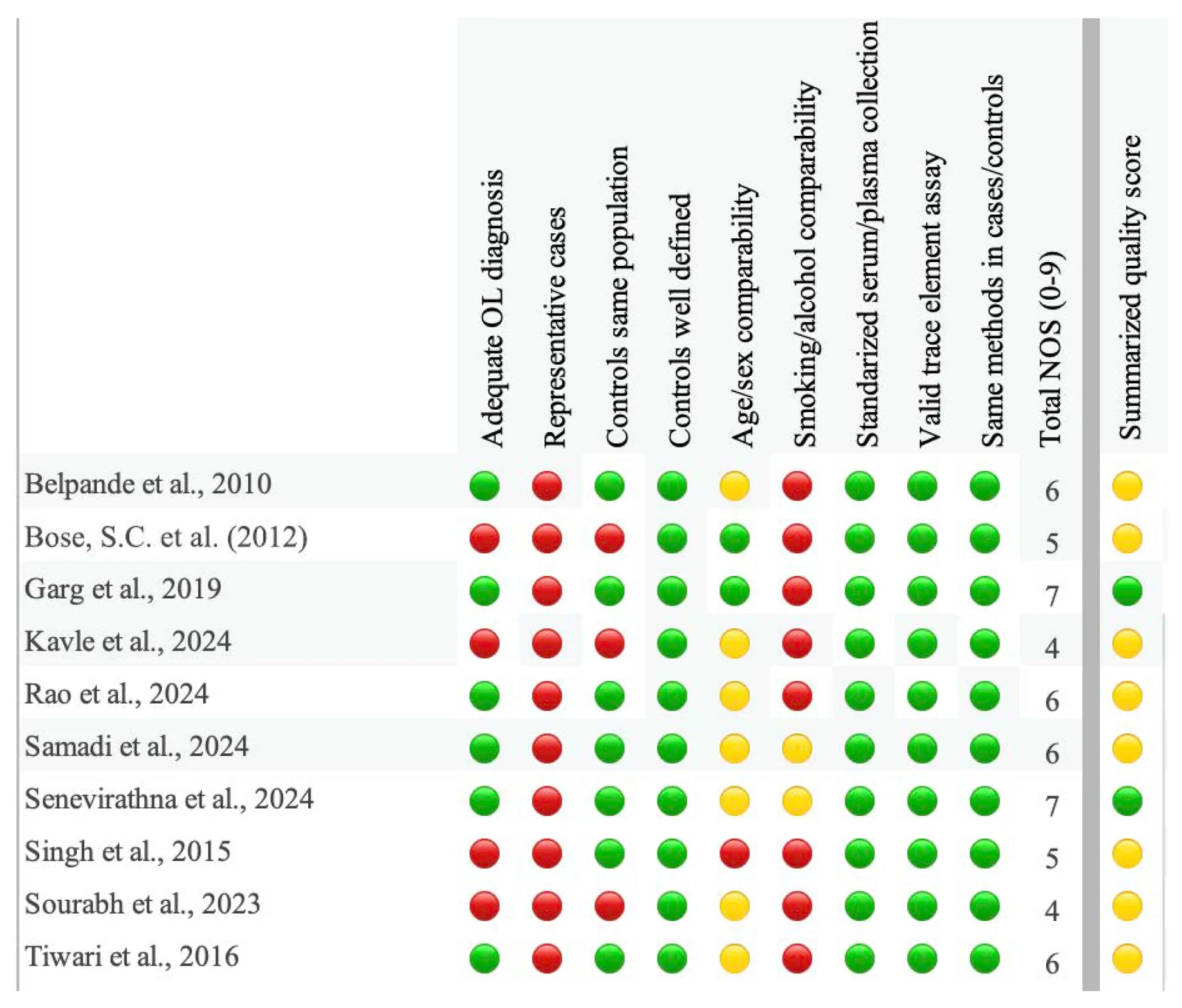
Risk of bias assessment [32,33,34,35,36,37,38,39,40,41].

All incorporated investigations adopted a case–control design, with oral leukoplakia diagnoses established clinically and, in the majority of instances, corroborated by histopathological examination. Methodological quality, as assessed using the Newcastle–Ottawa Scale, yielded scores spanning 4 to 7 (Table 2). According to our prespecified quality thresholds, eight studies (scoring 4–6) were categorized as being of moderate quality, while the remaining two studies (scoring 7–9) were deemed high quality; no study fell into the low-quality category (Figure 2).

### 3.3. Serum and Plasma Zinc and Copper Concentrations in Patients with Oral Leukoplakia

#### 3.3.1. Serum and Plasma Zinc Concentrations in Patients with Oral Leukoplakia

Eight studies comprising 431 participants, including 223 patients with oral leukoplakia and 208 healthy controls, were included in the meta-analysis. The random-effects REML model showed that patients with oral leukoplakia had significantly lower plasma or serum zinc concentrations than controls, with a large pooled effect size (Hedges’ g = −1.84, 95% CI: −3.25 to −0.43; z = −2.55, *p* = 0.01). Substantial between-study heterogeneity was observed (τ^2^ = 4.00; I^2^ = 96.85%; H^2^ = 31.79; Q(7) = 169.24, *p* < 0.001). Six studies reported significantly lower zinc concentrations in patients with oral leukoplakia, whereas Senevirathna et al. (2024) found no statistically significant difference and Samadi et al. (2024) reported significantly higher zinc concentrations in the oral leukoplakia group [32,33,34,35,37,38,39,40].

**Figure 3:**
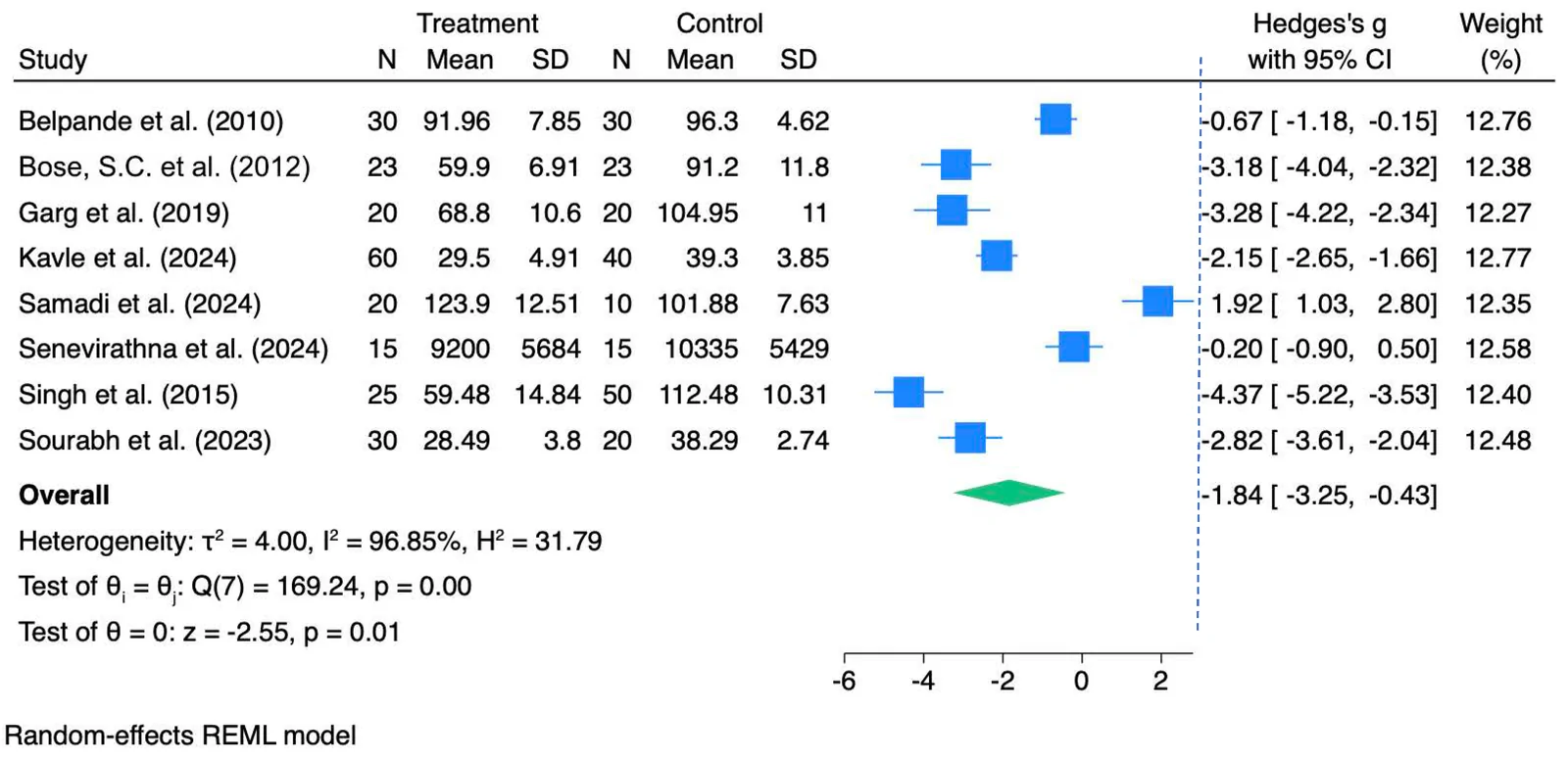
Random-effects REML model—plasma and serum zinc concentration in patients with oral leukoplakia and healthy controls [32,33,34,35,37,38,39,40].

Overall, the pooled analysis indicated reduced plasma or serum zinc concentrations in patients with oral leukoplakia (Figure 3).

#### 3.3.2. Serum and Plasma Copper Concentrations in Patients with Oral Leukoplakia

Nine studies comprising 449 participants, including 224 patients with oral leukoplakia and 225 healthy controls, were included in the meta-analysis of plasma or serum copper concentrations. The random-effects REML model demonstrated significantly higher copper concentrations in patients with oral leukoplakia than in controls, with a large pooled effect size (Hedges’ g = 1.20, 95% CI: 0.70–1.70; z = 4.68, *p* < 0.001). Considerable between-study heterogeneity was observed (τ^2^ = 0.48; I^2^ = 81.90%; H^2^ = 5.52; Q(8) = 35.58, *p* < 0.001). Seven studies reported significantly higher copper concentrations in patients with oral leukoplakia, whereas Rao et al. (2014) and Senevirathna et al. (2024) found no statistically significant between-group differences [32,34,35,36,37,38,39,40,41].

**Figure 4:**
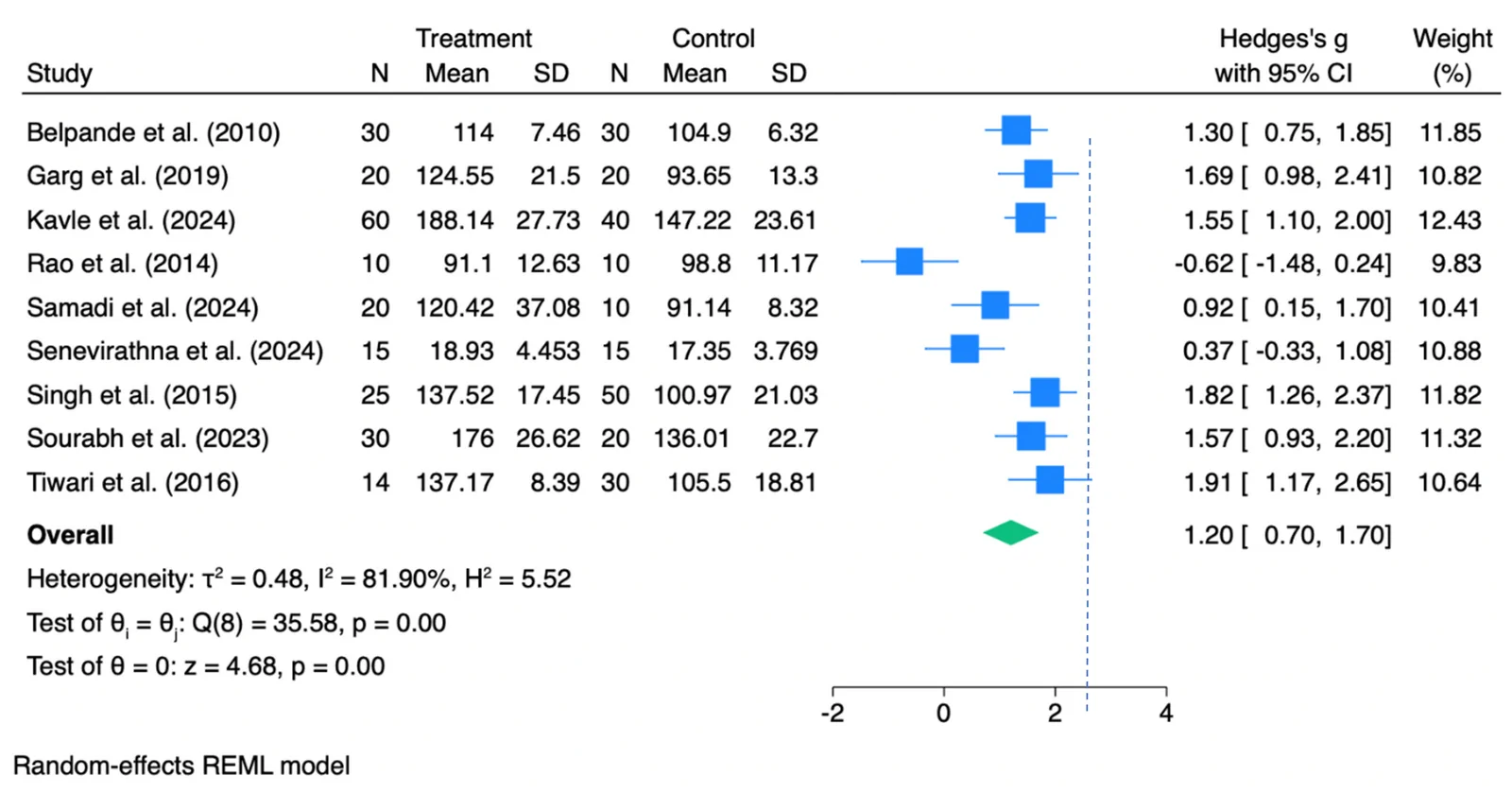
Random-effects REML model—plasma and serum copper concentration in patients with oral leukoplakia and healthy controls [32,34,35,36,37,38,39,40,41].

Overall, the pooled analysis indicated increased plasma or serum copper concentrations in patients with oral leukoplakia (Figure 4).

### 3.4. Serum and Plasma Zinc and Copper Concentrations According to the Grade of Epithelial Dysplasia in Oral Leukoplakia

Two studies evaluated serum copper and zinc concentrations according to the grade of epithelial dysplasia in oral leukoplakia. Samadi et al. (2024) included 20 patients, comprising 10 cases of mild, five of moderate, and five of severe dysplasia [37]. Serum copper concentrations increased progressively with dysplasia severity, from 89.50 ± 9.79 µg/dL in mild dysplasia to 131.26 ± 6.99 µg/dL in moderate dysplasia and 171.41 ± 21.91 µg/dL in severe dysplasia. A similar, although less pronounced, pattern was observed for serum zinc, with concentrations of 117.74 ± 11.25, 121.41 ± 8.28, and 138.71 ± 3.72 µg/dL in mild, moderate, and severe dysplasia, respectively.

Tiwari et al. (2016) assessed only serum copper concentrations and likewise reported higher values in patients with severe dysplasia than in those with moderate dysplasia [41]. The mean copper concentration was 133.18 ± 5.05 µg/dL in the moderate-dysplasia group (n = 10) and 147.14 ± 6.53 µg/dL in the severe-dysplasia group (n = 4).

Given the limited number of studies and participants available for this comparison, the exploratory analysis showed an increase in serum copper concentrations with increasing severity of epithelial dysplasia. Evidence regarding zinc was based on a single study and showed higher concentrations in severe dysplasia.

### 3.5. Subgroup Analysis According to the Analytical Method

Subgroup analyses were performed according to the analytical method used to determine serum or plasma copper and zinc concentrations, namely, inductively coupled plasma optical emission spectrometry (ICP-OES) and colorimetric assays.

**Figure 5:**
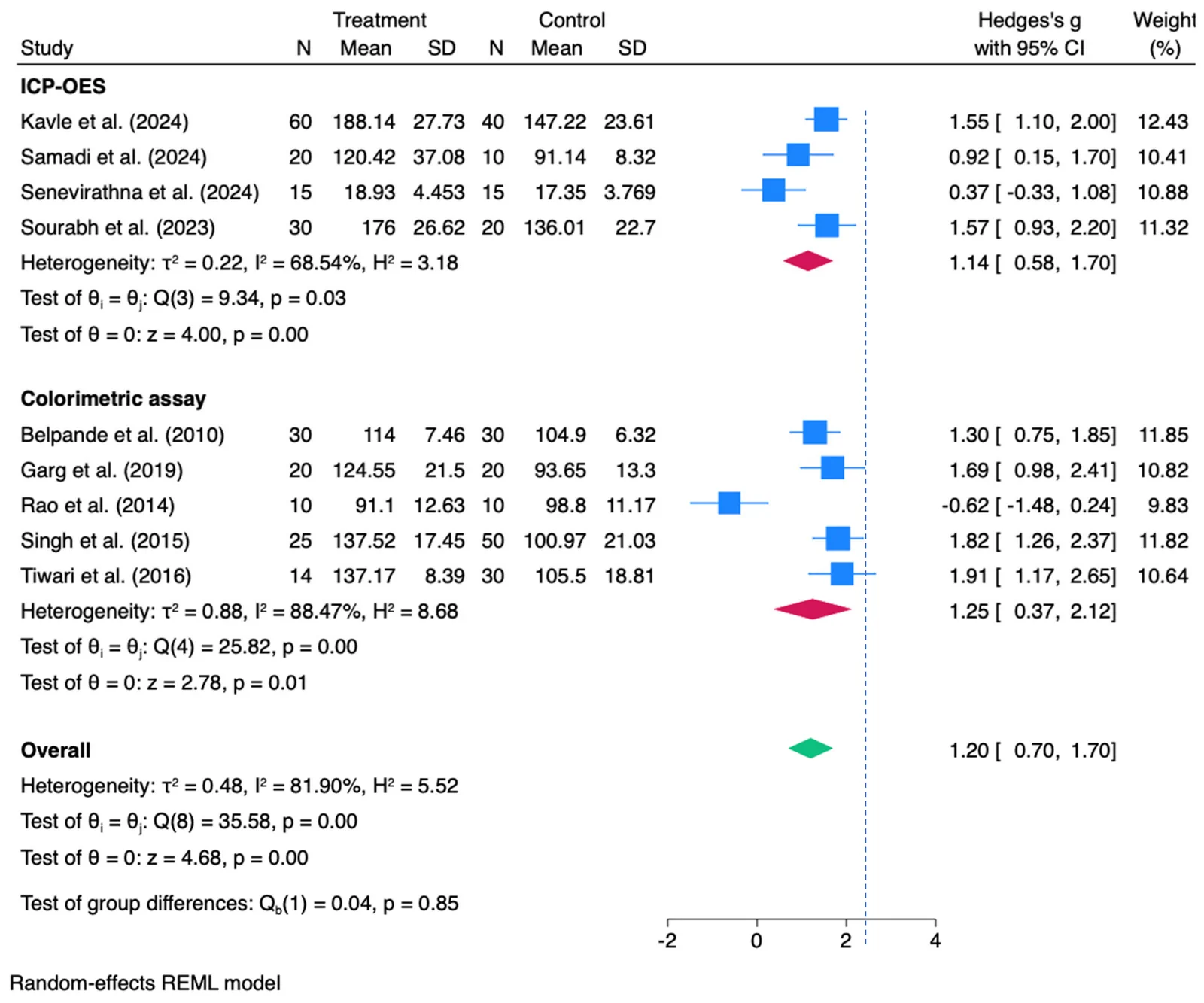
Random-effects REML model—subgroup analysis according to the analytical method for copper [32,34,35,36,37,38,39,40,41].

For copper, both analytical-method subgroups showed significantly higher concentrations in patients with oral leukoplakia than in healthy controls. In the ICP-OES subgroup, comprising four studies, the pooled effect size was large and statistically significant (Hedges’ g = 1.14, 95% CI: 0.58–1.70; z = 4.00, *p* < 0.001). Moderate-to-substantial heterogeneity was observed (τ^2^ = 0.22; I^2^ = 68.54%; Q(3) = 9.34, *p* = 0.03). Similarly, the five studies using colorimetric assays demonstrated significantly higher copper concentrations in the oral leukoplakia group (Hedges’ g = 1.25, 95% CI: 0.37–2.12; z = 2.78, *p* = 0.01), although heterogeneity was considerable (τ^2^ = 0.88; I^2^ = 88.47%; Q(4) = 25.82, *p* < 0.001). There was no significant difference between the two analytical-method subgroups (Q_b(1) = 0.04, *p* = 0.85), indicating that the method of copper determination did not significantly modify the pooled effect (Figure 5).

For zinc, the results differed numerically between the two subgroups. Among the four studies using ICP-OES, the pooled effect did not indicate a statistically significant difference between patients with oral leukoplakia and controls (Hedges’ g = −0.83, 95% CI: −2.90 to 1.25; z = −0.78, *p* = 0.44). This subgroup was characterized by very high heterogeneity (τ^2^ = 4.35; I^2^ = 97.21%; Q(3) = 86.13, *p* < 0.001). In contrast, the four studies using colorimetric assays showed significantly lower zinc concentrations in patients with oral leukoplakia (Hedges’ g = −2.85, 95% CI: −4.42 to −1.27; z = −3.54, *p* < 0.001), also with substantial heterogeneity (τ^2^ = 2.41; I^2^ = 94.06%; Q(3) = 69.70, *p* < 0.001).

**Figure 6:**
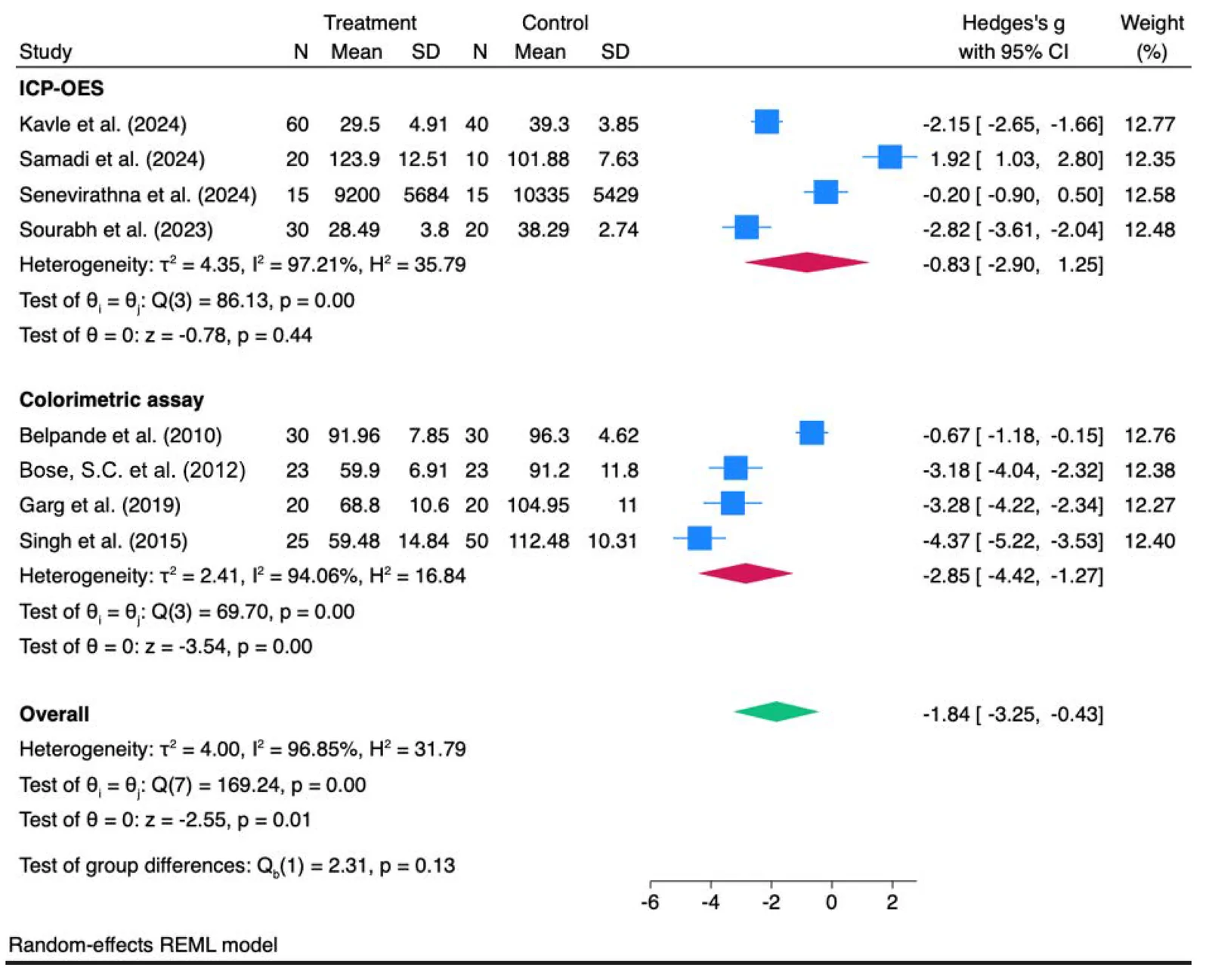
Random-effects REML model—subgroup analysis according to the analytical method for zinc [32,33,34,35,37,38,39,40].

Nevertheless, the formal test for subgroup differences was not statistically significant (Q_b(1) = 2.31, *p* = 0.13). Thus, although the pooled effect reached statistical significance only in studies using colorimetric assays, there was insufficient evidence that the analytical method significantly influenced the estimated association between oral leukoplakia and zinc concentrations (Figure 6).

### 3.6. Leave-One-out Sensitivity Analysis

The leave-one-out sensitivity analysis confirmed the robustness of the pooled findings for both copper and zinc.

**Figure 7:**
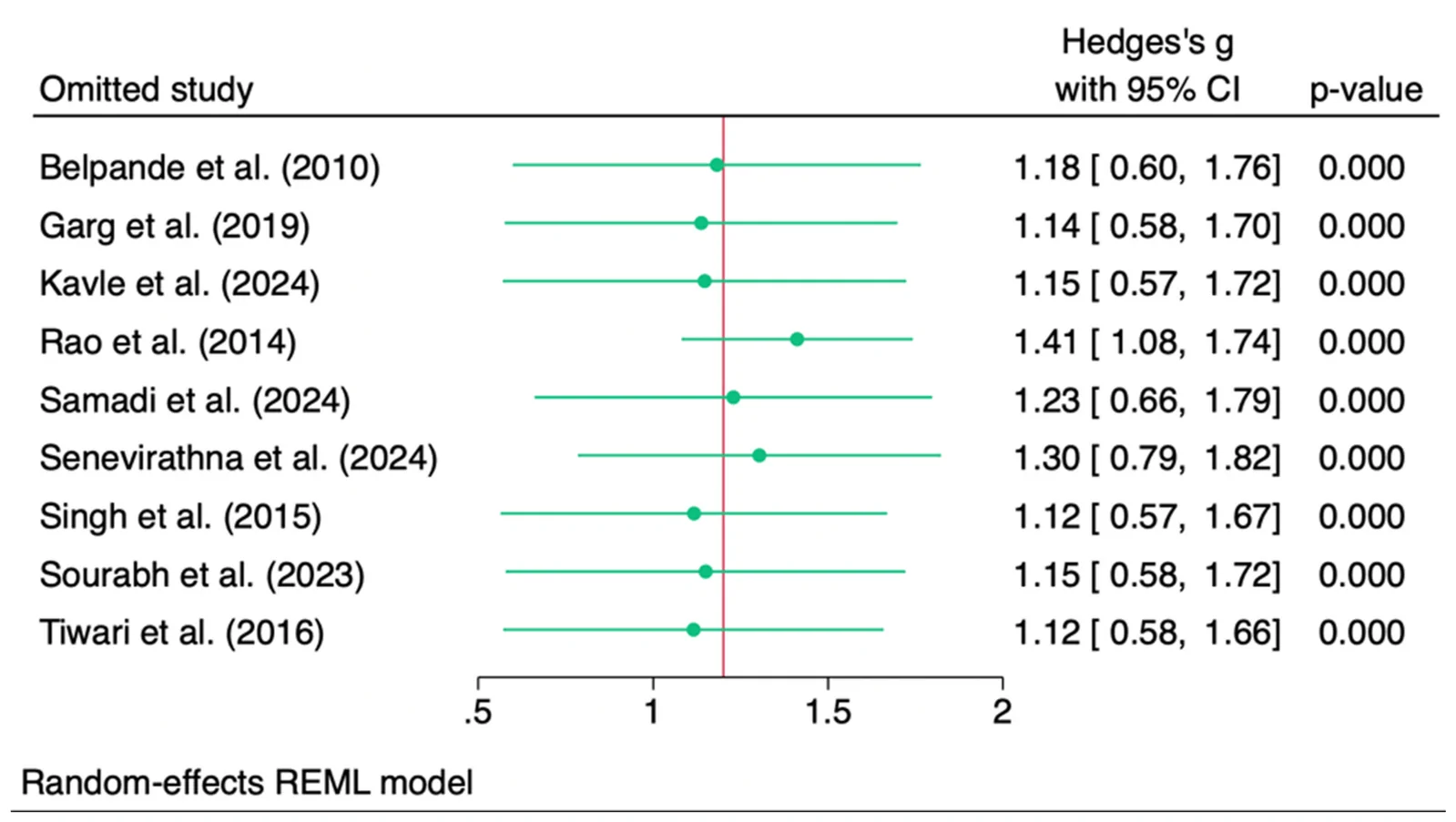
Random-effects REML model—leave-one-out sensitivity analysis for copper [32,34,35,36,37,38,39,40,41].

For copper, omission of each study in turn produced pooled effect estimates ranging from Hedges’ g = 1.12 to 1.41. All recalculated effects remained statistically significant (all *p* < 0.001), and none of the corresponding 95% confidence intervals crossed the null value. The largest pooled effect was observed after excluding Rao et al. (2014) (Hedges’ g = 1.41, 95% CI: 1.08–1.74), whereas the smallest estimates were obtained after excluding Singh et al. (2015) or Tiwari et al. (2016) (Hedges’ g = 1.12) [36,39,41]. These findings indicate that the higher copper concentrations observed in patients with oral leukoplakia were not driven by any single study (Figure 7).

**Figure 8:**
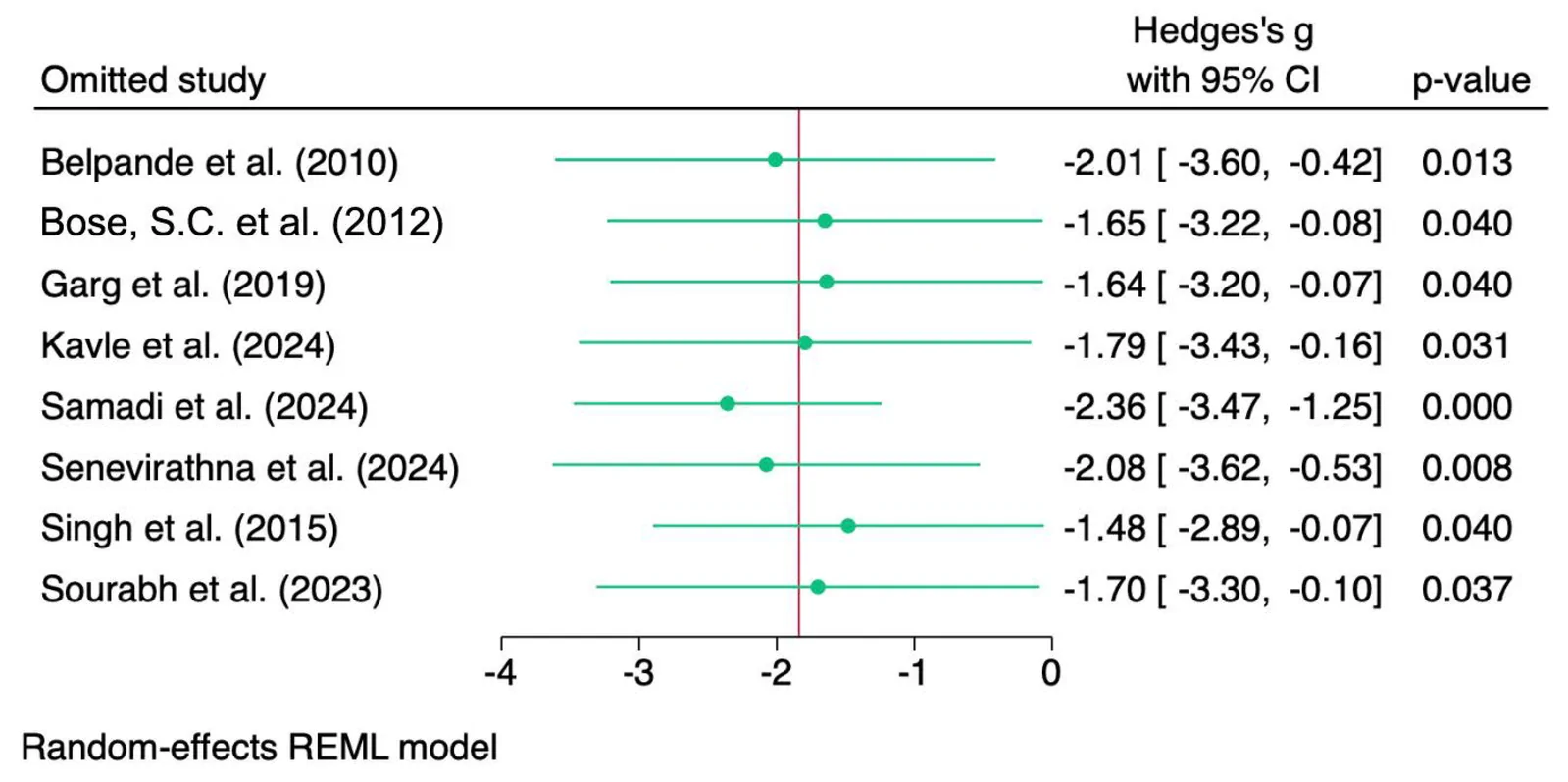
Random-effects REML model—leave-one-out sensitivity analysis for zinc [32,33,34,35,37,38,39,40].

Similarly, the pooled association for zinc remained negative and statistically significant after the sequential exclusion of each study. The recalculated effect sizes ranged from Hedges’ g = −2.36 to −1.48, with all 95% confidence intervals remaining below zero (*p* values ranging from <0.001 to 0.040). Exclusion of Samadi et al. (2024) resulted in the largest reduction in zinc concentrations among patients with oral leukoplakia (Hedges’ g = −2.36, 95% CI: −3.47 to −1.25; *p* < 0.001), whereas exclusion of Singh et al. (2015) produced the smallest effect estimate (Hedges’ g = −1.48, 95% CI: −2.89 to −0.07; *p* = 0.040) [37,39]. Overall, no individual study materially altered the direction or statistical significance of the pooled zinc effect (Figure 8).

### 3.7. GRADE Assessment

The overall certainty of evidence regarding serum or plasma zinc and copper alterations in patients with oral leukoplakia was rated as very low according to the GRADE approach [29]. The evidence originated exclusively from observational case–control or cross-sectional studies and was downgraded by one level for serious risk of bias, as most studies were of moderate methodological quality, and by two levels for very serious inconsistency because of considerable and largely unexplained between-study heterogeneity. No downgrading was applied for indirectness or imprecision, as the studies directly addressed the review question and the pooled confidence intervals did not cross the null value. Publication bias could not be formally evaluated because fewer than ten studies contributed to each meta-analysis [31]. Despite the large pooled effect sizes and the stability of the findings in leave-one-out sensitivity analyses, the evidence was not upgraded because of residual methodological limitations and substantial heterogeneity (Supplementary File S2).

## 4. Discussion

Oral leukoplakia is a clinical diagnosis of exclusion that encompasses white lesions of the oral mucosa that cannot be attributed to any other defined disease or disorder. It remains one of the most commonly reported oral potentially malignant disorders, and its clinical significance is primarily related to the relatively high risk of transformation into oral squamous cell carcinoma [42,43]. Although established risk factors, such as tobacco exposure, alcohol consumption, and other chronic irritants, are well documented, the biological mechanisms underlying the development and progression of OL have not yet been fully elucidated [44,45,46].

Increasing attention has been paid to oxidative stress in this context, as excessive production of reactive oxygen species (ROS), accompanied by impaired antioxidant defense mechanisms, may lead to lipid, protein, and DNA damage, thereby promoting cellular instability and the progression of premalignant lesions [47,48].

Previous studies suggest that disturbances in oxidative–antioxidative balance and trace element homeostasis in OL may occur at both local and systemic levels. Salivary studies are particularly relevant to the local oral microenvironment because saliva remains in direct contact with oral lesions and may reflect local inflammatory, oxidative, and metabolic processes occurring within the oral mucosa [49,50,51]. In contrast, measurements performed in serum or plasma may help determine whether OL is associated with more widespread biological alterations, including systemic inflammatory responses, environmental exposures, and disturbances in systemic redox homeostasis [52,53,54].

The distinction between local and systemic biomarkers is important for the interpretation of these findings. Although leukoplakia develops locally within the oral mucosa, the processes contributing to its initiation and progression may also be modulated by systemic factors. From this perspective, the assessment of serum or plasma zinc and copper concentrations complements previous observations derived from salivary biomarkers by extending the analysis beyond the local oral microenvironment toward potential systemic alterations associated with OL.

The present meta-analysis demonstrated that patients with oral leukoplakia had significantly lower serum/plasma zinc concentrations than healthy controls (SMD = −1.84, 95% CI: −3.25 to −0.43; z = −2.55, *p* = 0.01). This finding indicates a substantial difference in circulating Zn levels between patients with OL and healthy individuals. Interestingly, in a qualitative analysis considering histopathological severity, Samadi et al. (2024) reported a gradual increase in zinc concentration with increasing severity of epithelial dysplasia, from 117.74 ± 11.25 µg/dL in mild dysplasia to 138.71 ± 3.72 µg/dL in severe dysplasia [37]. However, this observation should be interpreted with particular caution, as it was derived from a single study and therefore does not provide sufficient evidence to establish a reliable relationship between the severity of epithelial dysplasia and circulating Zn concentrations. Moreover, the study by Samadi et al. (2024) [37] was the only study included in the meta-analysis in which overall Zn concentrations were higher in the OL group than in healthy controls (SMD = 1.92). This isolated finding is inconsistent with the results of the remaining studies and should therefore be interpreted cautiously.

Zinc is an essential trace element that plays a fundamental role in maintaining redox homeostasis, cellular structural stability, and the regulation of gene expression [55,56,57,58]. As a non-enzymatic antioxidant, it protects protein sulfhydryl groups against oxidation and constitutes an essential component of copper/zinc superoxide dismutase (Cu/Zn-SOD), a key enzyme responsible for the dismutation of superoxide radicals [57,59,60,61,62,63]. Moreover, Zn competes with transition metals such as copper and iron for binding sites on cellular membranes and proteins, thereby limiting the formation of highly reactive hydroxyl radicals through Fenton- and Haber–Weiss-type reactions [64,65,66]. Reduced serum zinc concentrations in patients with OL may be associated with impaired systemic antioxidant defenses and greater tissue susceptibility to oxidative damage induced by carcinogenic exposures, such as tobacco smoke or alcohol [67,68,69]. However, the observational nature of the available evidence does not allow determination of whether reduced zinc concentrations contribute to these processes or occur as a consequence of them. Decreased Zn concentrations may therefore also represent a secondary phenomenon. Increased ROS production within a developing lesion could contribute to the depletion or altered turnover of antioxidant systems in which zinc plays an important biological role, thereby affecting systemic zinc availability. Exploratory findings of increased Zn concentrations at more advanced stages of dysplasia, reported in isolated studies, may hypothetically reflect compensatory mechanisms involving mineral mobilization from tissue stores and a consequent increase in systemic zinc levels.

According to the clinical studies included in the analysis, patients diagnosed with oral leukoplakia exhibited significantly higher serum/plasma copper concentrations than healthy controls (SMD = 1.20, 95% CI: 0.70–1.70; z = 4.68, *p* < 0.001). This statistically significant result indicates a marked increase in circulating Cu concentrations in patients with OL. Samadi et al. (2024) [37] additionally evaluated the degree of dysplastic changes in 20 patients and observed a progressive increase in copper levels, from 89.50 ± 9.79 µg/dL in mild dysplasia, through 131.26 ± 6.99 µg/dL in moderate dysplasia, to 171.41 ± 21.91 µg/dL in severe dysplasia. These findings suggest that copper concentrations may increase with the severity of dysplastic progression in OL [37]. Similar observations were reported by Tiwari et al. (2016). Among 10 patients with moderate dysplasia, the mean copper concentration was 133.18 ± 5.05 µg/dL, whereas in four patients with severe dysplasia, a higher mean concentration of 147.14 ± 6.53 µg/dL was reported [41]. Taken together, these two studies suggest that increasing Cu concentrations with increasing severity of dysplasia may represent a reproducible pattern, although the limited sample sizes warrant cautious interpretation.

Copper plays an important role in cellular metabolism as a cofactor for several enzymes, including cytochrome c oxidase (CcO), as well as in antioxidant defense mechanisms through its involvement in SOD activity. Accordingly, increased copper availability could be consistent with the elevated metabolic and energetic demands of highly proliferative cells, although the present observational findings do not establish such a mechanism [70,71,72]. Several mechanisms may potentially contribute to increased circulating copper levels. One involves Fenton and Haber–Weiss reactions, in which hydrogen peroxide is converted into highly reactive hydroxyl radicals in the presence of transition metals, including copper. Copper ions have been reported to exhibit particularly strong pro-oxidant properties in the generation of ROS [73,74,75]. Tobacco and areca exposure have also been suggested as potential exogenous factors that may influence copper availability in this context [33,76,77].

Another potentially relevant mechanism involves ceruloplasmin. During inflammatory or neoplastic processes, hepatic production of ceruloplasmin, an acute-phase protein and the major copper-transporting protein in the circulation, may increase and could thereby contribute to elevated circulating copper concentrations [34,76,78,79]. At the same time, SOD represents one of the major antioxidant enzymes responsible for the neutralization of reactive oxygen species, and copper is essential for the activity of the Cu/Zn-dependent form of SOD [71]. However, some studies have demonstrated reduced SOD activity or expression in malignant cells compared with healthy cells, suggesting impairment of cellular antioxidant defense mechanisms [80]. Taken together, these mechanisms provide possible biological explanations for the observed differences in Cu concentrations, but their contribution cannot be established from the available observational evidence.

Copper may also contribute to disease progression through its involvement in angiogenesis. By promoting the activity of angiogenic mediators, including VEGF, TNF-α, IL-1, and b-FGF, copper may facilitate neovascularization and thereby create conditions favorable to the progression of dysplastic lesions toward malignancy [14,79,81,82]. This mechanism could hypothetically contribute to the observed increase in Cu concentrations with increasing severity of epithelial dysplasia.

In the studies included in the present analysis, zinc and copper concentrations were determined using colorimetric assays or inductively coupled plasma-optical emission spectrometry (ICP-OES).

Colorimetric methods are based on a chemical reaction between the analyte and a specific reagent, resulting in the formation of a colored complex whose intensity is proportional to the concentration of the measured element. Their main advantages include simplicity, rapidity, low cost, and broad availability; however, these methods may be susceptible to analytical interference and have limited specificity [83,84,85].

In contrast, ICP-OES provides greater sensitivity and enables simultaneous determination of multiple elements through measurement of characteristic radiation emitted by atoms excited in plasma [83,86,87,88]. Nevertheless, the method is not without limitations, including sample drift, non-ideal detection limits, and potential inaccuracies in analyte identification or quantification [89,90,91]. In addition, ICP-OES requires highly specialized and relatively expensive instrumentation [92]. Despite these methodological differences, subgroup analyses revealed no statistically significant differences according to the analytical method used. This finding suggests that there is currently no evidence that the measurement technique substantially influenced the overall effect estimates. However, the limited statistical power of subgroup analyses resulting from the relatively small number of available studies should be taken into account [93,94].

Sensitivity analyses showed that exclusion of individual studies did not substantially alter either the magnitude or the direction of the pooled effect estimates, indicating that the meta-analytic findings were relatively stable and not driven by any single study. Furthermore, preservation of the same direction of effect following sequential study exclusion supports the consistency and robustness of the observed associations [95,96].

Assessment of publication bias was not feasible because of the small number of available studies. According to methodological recommendations, funnel plots and Egger’s test should be interpreted cautiously and are generally recommended when at least 10 studies are available, because their statistical power is limited when fewer studies are included, making reliable interpretation difficult [97,98].

The risk-of-bias assessment using the Newcastle–Ottawa Scale demonstrated an overall moderate level of methodological quality among the included studies [28]. Of all studies evaluated, eight were classified as having moderate methodological quality. Only two studies fulfilled the criteria for high methodological quality.

The main factor lowering the methodological assessment was the criterion concerning the representativeness of cases, as participants were recruited exclusively from research institutes or hospital-based centers. Another important limitation concerned comparability with respect to smoking and alcohol consumption. In most studies, the control groups consisted of non-smokers and non-drinkers, in contrast to the OL groups, or no information regarding these exposures was provided. Comparability in terms of age and sex was also only moderate. In the majority of studies, results were not stratified according to sex, or mean age with standard deviation was not reported separately for patients with leukoplakia; instead, these data were provided only for the broader group of oral potentially malignant disorders (OPMDs).

All of these factors influenced the overall assessment of methodological quality. Although none of the included studies was classified as being of low methodological quality and all received at least 4 points, the findings should nevertheless be interpreted with caution. Importantly, in four of the included studies, the diagnostic criteria used to identify OL were not clearly reported. This represents a relevant methodological limitation, as the diagnosis of OL should not be based on histopathological findings alone. Oral leukoplakia is primarily a clinical diagnosis of exclusion, established after other definable white lesions and potential local causative factors have been excluded. Histopathological examination provides important complementary information, particularly regarding the presence and severity of epithelial dysplasia, but even dysplastic changes are not specific to OL and may occur in lesions associated with identifiable causes, such as frictional keratosis [2]. Therefore, reliable identification of OL in clinical studies requires appropriate clinical assessment, including exclusion of potential causative factors, in addition to clearly reported histopathological evaluation where performed. Incomplete reporting of these diagnostic procedures may introduce heterogeneity and affect the comparability of the included populations.

A degree of selection bias and limited representativeness of the available data cannot be excluded, as has also been emphasized in studies investigating the prevalence of OPMDs [99,100,101]. Additional methodological limitations inherent to case–control designs, including the potential for overestimation of associations, have previously been discussed in the literature and should be considered when interpreting the present findings. These limitations should also inform the design of future studies in this field [102].

The GRADE assessment indicated that the overall certainty of evidence was very low, largely because of the characteristics of the available studies. All included studies were observational in design, either case–control or cross-sectional, and several exhibited methodological limitations, including incomplete reporting of participant characteristics and insufficient information regarding pre-analytical procedures. These factors may have introduced selection bias, confounding, and measurement error. However, the most important limitation was the substantial heterogeneity of the results, particularly in the analysis of zinc concentrations (I^2^ = 96.85%). Despite a generally consistent direction of the observed differences, effect sizes varied considerably across studies, and neither subgroup analyses nor sensitivity analyses were able to explain this variability. As noted above, publication bias could not be formally assessed because of the limited number of available studies, further reducing confidence in the pooled estimates. Consequently, these findings should be interpreted cautiously and require confirmation in future, better-designed studies.

The principal contribution of the present study is the collection and quantitative synthesis of previously scattered evidence regarding serum or plasma zinc and copper concentrations in patients with OL. Previous studies evaluating zinc and copper concentrations in biological samples from patients with OL have yielded inconsistent findings, providing a rationale for their quantitative synthesis [25,52]. The present meta-analysis demonstrates a recurring pattern characterized by lower zinc concentrations and higher copper concentrations in patients with OL compared with healthy individuals. Such a profile may be associated with alterations in zinc and copper homeostasis accompanying OL and may reflect biological processes related to oxidative stress, inflammatory responses, and dysregulation of antioxidant mechanisms [23,103,104]. However, the observational nature of the available evidence does not allow these alterations to be interpreted as contributing to the development or progression of OL. Moreover, the Zn/Cu ratio was not directly analyzed in the present study; therefore, any interpretation concerning an imbalance of the Zn/Cu axis remains hypothetical. The substantial heterogeneity of the findings, particularly for zinc, further indicates that the observed associations require confirmation before any diagnostic or prognostic significance can be considered.

The present findings also fit within the broader body of research investigating the potential role of antioxidant-based interventions in OL. A previous review of randomized clinical trials evaluating medical and complementary interventions for OL found no evidence that any currently available treatment effectively prevents the development of oral cancer, although individual studies have reported clinical regression of lesions following the use of selected compounds, including vitamin A, retinoic acid, beta-carotene, and lycopene [105]. In a randomized trial evaluating lycopene, two doses, 8 mg and 4 mg daily, were compared with placebo, with more favorable clinical responses observed in the lycopene groups [106]. Similarly, studies investigating curcumin have suggested that clinical improvement may occur in a subset of patients with OL, including a study in which 3.6 g of curcumin was administered daily for 6 months [107]. A more recent study comparing curcumin, lycopene, and their combination also reported clinical improvement following these nutraceutical interventions, although its design did not allow their efficacy to be evaluated against placebo [108]. Interestingly, photodynamic therapy, which has also been successfully applied in the treatment of OL, was shown in patients with oral lichen planus to reduce selected markers of oxidative stress. This observation raises the possibility that, in addition to its established local phototoxic effects, PDT may also exert therapeutic activity by modulating oxidative stress, a mechanism that may be relevant to the pathogenesis of OL [109,110]. Collectively, these findings suggest potential therapeutic value of antioxidant interventions, but the available evidence remains insufficient to support their use as a standard treatment for OL.

Although the reduced zinc concentrations observed in patients with OL may suggest an association between zinc status and impaired antioxidant defense, the currently available evidence does not support isolated zinc supplementation as an established treatment for OL. Existing interventional studies have predominantly evaluated antioxidant therapies or multicomponent preparations rather than zinc as a stand-alone intervention [111]. Therefore, zinc supplementation may be considered for the correction of a documented deficiency or as a hypothesis to be investigated in future clinical trials, but it should not currently be regarded as a specific treatment for oral leukoplakia.

Copper should be interpreted differently. Given that the present meta-analysis demonstrated higher circulating copper concentrations in patients with OL, copper supplementation currently lacks both biological and clinical justification in this context.

### Limitations

Several limitations of this meta-analysis should be acknowledged. Most importantly, the available evidence was derived entirely from observational studies and was characterized by substantial between-study heterogeneity, particularly for zinc. The predominantly case–control design of the included studies precludes causal inference and does not allow determination of whether altered zinc and copper concentrations contribute to OL development or represent a consequence of the disease process. Considerable heterogeneity was observed for both zinc and copper and remained largely unexplained despite subgroup and sensitivity analyses. In addition, the relatively small number of eligible studies limited the statistical power of subgroup analyses and restricted the possibility of reliably identifying sources of heterogeneity.

Potential confounding also remains an important concern, as several studies incompletely reported or inadequately matched participants with respect to smoking, alcohol consumption, age, sex, and other factors potentially affecting trace element status, such as diet, nutritional status, or supplementation. Methodological variability in sample type, pre-analytical procedures, storage conditions, and analytical techniques may have additionally contributed to the observed heterogeneity.

An additional limitation concerns the diagnostic characterization of OL, as the diagnostic criteria were not clearly reported in four of the included studies. Given that OL is primarily a clinical diagnosis of exclusion, insufficient reporting of the clinical diagnostic process may have affected the comparability of the study populations. Furthermore, detailed histopathological information regarding the included lesions was generally limited. Oral leukoplakia may encompass a broad histopathological spectrum, ranging from epithelial hyperplasia or hyperkeratosis without dysplasia to varying degrees of epithelial dysplasia and, in some cases, previously unrecognized invasive carcinoma. Such histopathological heterogeneity may potentially influence Zn and Cu concentrations and therefore represents an additional source of variability that could not be adequately explored in the present meta-analysis. Moreover, the evidence was geographically concentrated, with most studies originating from South and Southeast Asia, which may limit the generalizability of the findings to other populations.

Data regarding the relationship between trace element concentrations and the severity of epithelial dysplasia were scarce, and no longitudinal evidence was available to determine whether zinc or copper levels are associated with subsequent malignant transformation. The limited availability of histopathological grading also precluded a reliable assessment of whether Zn or Cu concentrations vary according to the severity of epithelial dysplasia. The Zn/Cu ratio could not be evaluated because of insufficient reporting in the primary studies. Publication bias could not be formally assessed because fewer than ten studies were available for each meta-analysis. Finally, only a small number of eligible studies were identified through citation searching outside the major electronic databases, which may indicate that additional unpublished or poorly indexed evidence was not captured.

Therefore, the present findings should be interpreted cautiously and require confirmation in larger, prospectively designed, geographically diverse studies with standardized diagnostic criteria, detailed histopathological characterization, standardized analytical procedures, and better control of potential confounding factors.

## 5. Conclusions

This systematic review and meta-analysis of observational studies indicates that patients with oral leukoplakia have lower serum/plasma zinc concentrations and higher copper concentrations than healthy controls. However, these findings were derived from highly heterogeneous studies and should be interpreted as differences between groups rather than evidence of a causal, metabolic, or pathogenic role of altered trace element levels. The current evidence is insufficient to determine whether serum/plasma Zn and Cu concentrations play a specific role in the cellular metabolism or pathogenesis of OL. The observed differences may reflect broader alterations accompanying the disease, but their biological significance remains uncertain. The substantial heterogeneity, particularly for zinc, together with the very low certainty of evidence, further limits the strength and generalizability of these conclusions.

Further well-designed prospective studies with standardized analytical methods and adequate control of confounding factors are required to clarify the biological significance of zinc and copper alterations in oral leukoplakia and to determine whether they have any diagnostic or prognostic relevance.

## Figures and Tables

**Table 1:** Inclusion and exclusion criteria shown using the PECO framework.

Parameter	Inclusion Criteria	Exclusion Criteria
Population (P)	Patients of any age or gender	-
Exposure (E)	Presence of at least one OL lesion in the oral cavity	Studies not reporting OL lesions
Comparison (C)	Systemically healthy controls without any OPMD’s lesion	Studies without a healthy controls
Outcomes (O)	Serum/plasma trace elements: copper and/or zinc	Studies assessing only non-serum or non-plasma samples (saliva, whole blood, tissue, gingival crevicular fluid); studies without serum/plasma trace elements, purely qualitative results or data insufficient for effect size calculation.
Study Design	Case–control or cross-sectional studies with both OL and control groups	Systematic reviews, narrative reviews, meta-analyses, case reports/series, conference abstracts, editorials, letters, theses; non-comparative designs; works not published in English; non-human or in vitro studies.

**Table 2:** Main characteristics of the case–control studies included in the review, presenting sample size, demographic data, assessed trace elements, and methodological quality scores.

			Sample Size (M/F)		Age (Mean ± SD; Years)			
Author	Country	Study Design	OL	HC	OL	HC	Markers	Quality score
Belpande et al. (2010) [32]	India	CC	30 (ND)	30 (ND)	47.33 ± 14.46	37.53 ± 13.46	Copper, Zinc	6
Bose et al. (2012) [33]	India	CC	23 (ND)	23 (ND)	ND	ND	Zinc	5
Garg et al. (2019) [34]	Malaysia	CC	20 (ND)	20 (ND)	ND	ND	Copper, Zinc	7
Kavle et al. (2024) [35]	India	CC	60 (ND)	40 (ND)	ND	ND	Copper, Zinc	4
Rao et al. (2014) [36]	India	CC	10 (ND)	10 (ND)	ND	ND	Copper	6
Samadi et al. (2024) [37]	India	CC	20 (ND)	10 (ND)	ND	59.66 ± 8.26	Copper, Zinc	6
Senevirathna et al. (2024) [38]	Sri Lanka	CC	15 (9/6)	15 (9/6)	62 ± 13.111	37 ± 11.247	Copper, Zinc	7
Singh et al. (2015) [39]	India	CC	25 (ND)	50 (ND)	ND	ND	Copper, Zinc	5
Sourabh et al. (2023) [40]	India	CC	30 (27/3)	20 (15/5)	46.97 ± 2.56	48.90 ± 2.84	Copper, Zinc	4
Tiwari et al. (2016) [41]	India	CC	14 (ND)	30 (17/13)	ND	33.26 ± 7.72	Copper	6

CC—case–control study; HC—healthy control group; ND—no data.

**Table 3:** Serum/plasma sampling and pre-analytical procedures used in the included studies.

Author	OL Diagnostic Method	Serum/Plasma	Sample Volume	Coagulation Time	Sample Preparation	Analytical Method
Belpande et al. (2010) [32]	Histopathological	Serum	3 mL	30 min	Centrifuged for 15 min at 3000 rpm, then stored at 2 to 8 °C for 5 days	Colorimetric assay
Bose et al. (2012) [33]	ND	Serum	5 mL	ND	Centrifuged at 4 °C then stored at 20 °C	Colorimetric assay
Garg et al. (2019) [34]	Clinical and histopathological	Plasma	5 mL	ND	Centrifuged at 2000 rpm, then frozen at −10 °C	Colorimetric assay
Kavle et al. (2024) [35]	ND	Serum	5 mL	120 min	Centrifuged for 10 min at 3000 rpm, then frozen at −20 °C	ICP-OES
Rao et al. (2014) [36]	Clinical and histopathological	Serum	2 mL	ND	Centrifuged	Colorimetric assay
Samadi et al. (2024) [37]	Histopathological	Serum	4–5 mL	60 min	Centrifuged for 5 min at 3000 rpm, then frozen at −65 °C	ICP-OES
Senevirathna et al. (2024) [38]	Clinical or histopathological	Serum	ND	ND	ND	ICP-OES
Singh et al. (2015) [39]	ND	Serum	ND	ND	ND	Colorimetric assay
Sourabh et al. (2023) [40]	ND	Serum	5 mL	120 min	Centrifuged for 10 min at 3000 rpm, then frozen at −20 °C	ICP-OES
Tiwari et al. (2016) [41]	Clinical and histopathological	Serum	5 mL	60 min	Centrifuged for 10 min at 2000 rpm	Colorimetric assay

ND—no data; ICP-OES—inductively coupled plasma optical emission spectrometry.

**Table 4:** Pooled results of the random/fixed effects meta-analyses for serum copper and zinc in patients with oral leukoplakia compared with healthy controls, including number of contributing studies and participants, standardized mean differences with 95% confidence intervals, and heterogeneity statistics.

				Effect Size (OL vs. HC)			Heterogeneity			
Trace Element	n Studies	n OL	n HC	SMD (95% CI)	Z	*p*	Chi^2^	df	*p*	I^2^ (%)
Copper	9	224	225	1.20 (0.7; 1.7)	4.68	<0.0001	35.58	8	<0.001	81.90
Zinc	8	223	208	−1.84 (−3.25; −0.43)	−2.55	0.01	169.24	7	<0.001	96.85

## Data Availability

No new data were created or analyzed in this study. Data sharing is not applicable to this article.

## Supplementary Materials

The following supporting information can be downloaded at https://www.mdpi.com/article/10.3390/antiox15101312/s1, Supplementary File S1: PRISMA Checklist; Supplementary File S2: GRADE Evidence Profile.
