# Supplementary material for: Serum and Plasma Zinc and Copper Levels in Oral Leukoplakia: A Systematic Review and Meta-Analysis
Source: Antioxidants (Basel). 2026 Oct 12;15(10):1312. doi: 10.3390/antiox15101312 (PMC13700899; doi:10.3390/antiox15101312)
Supplement: Supplementary file 1 [file antioxidants-15-01312-s001.zip › antioxidants-4525069-Supplementary File S2.pdf]

Supplementary File S2 - GRADE Evidence Profile

This table summarizes the GRADE assessment for the overall body of evidence regarding differences in serum or plasma zinc and copper concentrations between patients with oral leukoplakia and healthy controls. The GRADE approach was applied to the evidence as a whole rather than separately to each trace element. Judgments were based on study design, the modified Newcastle-Ottawa Scale risk-of-bias assessment, pooled Hedges' g estimates, heterogeneity statistics (I^2), subgroup analyses according to the analytical method, and leave-one-out sensitivity analyses. Ten studies were considered overall; nine contributed to the copper meta-analysis and eight to the zinc meta-analysis. Only studies included in the quantitative synthesis were considered.

GRADE Domain	Judgment	Rationale	Impact on Certainty
Risk of Bias	Serious	All included studies had observational case-control or cross-sectional designs. According to the modified Newcastle-Ottawa Scale, two studies were classified as high quality, whereas eight were of moderate quality. Incomplete reporting of participant characteristics and group comparability, together with variability and occasional insufficient reporting of sample collection, storage, and pre-analytical procedures, may have introduced selection, confounding, and measurement bias.	Downgraded one level
Inconsistency	Very serious	Considerable heterogeneity was observed for copper (I^2 = 81.90%) and particularly for zinc (I^2 = 96.85%). Although most studies reported higher copper and lower zinc concentrations in patients with oral leukoplakia, the magnitude of the effects varied substantially. For zinc, individual studies included significant decreases, a non-significant difference, and one significant increase. Subgroup analyses according to the analytical method did not explain the heterogeneity and substantial variability remained despite the robustness of the leave-one-out analyses.	Downgraded two levels
Indirectness	Not serious	All studies directly compared patients with oral leukoplakia with healthy controls and assessed the outcomes of interest, namely serum or plasma copper and/or zinc concentrations. The populations, exposure, comparator and measured outcomes therefore closely matched the review question.	No downgrade
Imprecision	Not serious	Both pooled estimates were statistically significant, and their 95% confidence intervals did not cross the null value: Hedges' g = 1.20 (95% CI: 0.70-1.70) for copper and Hedges' g = -1.84 (95% CI: -3.25 to -0.43) for zinc. Although the confidence interval for zinc was relatively wide, the direction and statistical significance of both pooled effects were maintained throughout the leave-one-out sensitivity analyses.	No downgrade
Publication Bias	Undetected	Formal assessment of publication bias was not considered reliable because fewer than 10 studies contributed to each meta-analysis. Consequently, publication bias cannot be excluded.	No downgrade

Overall Certainty of Evidence: VERY LOW
